# Cortical plasticity between the pain and pain-free phases in patients with episodic tension-type headache

**DOI:** 10.1186/s10194-016-0698-6

**Published:** 2016-11-14

**Authors:** Bing Chen, Yuan He, Lei Xia, Li-Li Guo, Jin-Long Zheng

**Affiliations:** 1 Department of Neurology, Huai’an First People’s Hospital Affiliated to Nanjing Medical University, 223300, Beijing West Road 6#, Huai’an, Jiangsu Province People’s Republic of China; 2 Department of Gastrointestinal Surgery, Huai’an Hospital Affiliated to Xuzhou Medical College and Huai’an Second People’s Hospital, Huai’an, People’s Republic of China; 3 Department of Medical Imaging, Huai’an First People’s Hospital Affiliated to Nanjing Medical University, Huai’an, People’s Republic of China

**Keywords:** Episodic tension-type headache, Voxel-based morphometry, Gray matter density, Primary somatosensory cortex, Anterior cingulate cortex, Anterior insula

## Abstract

**Background:**

State-related brain structural alterations in patients with episodic tension-type headache (ETTH) are unclear. We aimed to conduct a longitudinal study to explore dynamic gray matter (GM) changes between the pain and pain-free phases in ETTH.

**Methods:**

We recruited 40 treatment-naïve ETTH patients and 40 healthy controls. All participants underwent brain structural scans on a 3.0-T MRI system. ETTH patients were scanned in and out of pain phases. Voxel-based morphometry analysis was used to determine the differences in regional gray matter density (GMD) between groups. Additional regression analysis was used to identify any associations between regional GMD and clinical symptoms.

**Results:**

ETTH patients exhibited reduced GMD in the bilateral primary somatosensory cortex, and increased GMD in the bilateral anterior cingulate cortex (ACC) and anterior insula for the in pain phase compared with the out of pain phase. The out of pain phase of ETTH patients exhibited no regions with higher or lower GMD compared with healthy controls. GMD in the left ACC and left anterior insula was negatively correlated with headache days. GMD in the left ACC was negatively correlated with anxiety and depressive symptoms in ETTH patients.

**Conclusions:**

This is the first study to demonstrate dynamic and reversible GMD changes between the pain and pain-free phases in ETTH patients. However, this balance might be disrupted by increased headache days and progressive anxiety and depressive symptoms.

## Background

Tension-type headache (TTH) is the most prevalent and the most neglected form of primary headache worldwide [1, 2]. Epidemiological studies reported that the 1-year prevalence of infrequent episodic TTH ranges from 48 to 63.5%, whereas the prevalence of frequent and chronic TTH is 21.6 to 34%, and 0.9 to 2%, respectively [3]. The infrequent and frequent episodic types can be combined under “episodic” TTH (ETTH) for pathophysiological purposes [4]. Although ETTH is generally considered to be less disabling than migraine, it has a greater socioeconomic impact [5, 6]. Patients with ETTH tend to go untreated unless headache symptoms are severe, which contributes to its progression [3, 4, 7]. The pathogenesis of ETTH remains incompletely understood. Peripheral pain mechanisms are most likely to predominate in ETTH, whereas involvement of central pain mechanisms in ETTH remains to be determined [4, 6, 8].

ETTH is characterized by a recurrence of pain and pain-free states. Recent neuroimaging studies from other cyclical recurrence of pain conditions, including episodic migraine, episodic cluster headache, and menstrual pain, demonstrated dynamic brain structural changes depending on the states of diseases. These findings suggest that this neural plasticity may be an important pathophysiological mechanism underlying these disorders [7, 9–13]. It is unclear, however, whether the state-related brain structural alterations actually exist in patients with ETTH.

Voxel-based morphometry (VBM) is a powerful analytical tool based on structural MRI data [14] that has been widely used to evaluate brain morphological alternations in different chronic pain syndromes [15, 16]. The only study using VBM in patients with chronic TTH demonstrated a significant gray matter (GM) decrease in pain-related brain structures, with which increasing headache duration was positively correlated [17]. These GM changes were considered the consequence of central sensitization in chronic TTH [17]. To our knowledge, no VBM study has been conducted in patients with ETTH to date.

The purpose of this study was to use whole-brain VBM analysis to longitudinally explore whether dynamic GM changes existed between pain and pain-free phases and to delineate possible relationships between GM changes and clinical variables in patients with ETTH. We hypothesized that ETTH exhibited state-related GM changes and that these regions might be involved in pain processing.

## Methods

### Subjects

An ETTH diagnosis was established according to the third edition (beta version) of the International Classification of Headache Disorders (ICHD) for ETTH [6]. Most of the patients were enrolled from our headache clinic. Some patients were enrolled from the local area by poster advertisement. Patients were 18 to 60 years old without a history of cognitive dysfunction. Only the treatment-naïve patients (at least three months) with ETTH were enrolled because previous evidence shows that treatment of chronic pain conditions [18, 19] and anti-inflammatory drugs [20] can affect brain morphometry. Exclusion criteria were as follows: any other type of primary or secondary headache or other pain disorders, systemic hypertension, diabetes, other systemic diseases, and previous history of head trauma, other neurologic diseases or psychiatric co-morbidities. Conventional MR images were evaluated to exclude participants with gross brain abnormalities. Finally, we enrolled 40 consecutive treatment-naïve patients with ETTH for the study. The ETTH patients were scanned twice during the pain and pain-free periods, separately. Patients were considered in the pain phase when they were experiencing acute headache attacks. Patients who were attack-free at least 3 days before and after the scans were considered to be in the pain-free phase [9]. The patients were not allowed to take any analgesic drugs until the end of the study unless they could not bear the headache. The visual analog scale (VAS) [21] was used to evaluate the rating of pain intensity during attacks in patients with ETTH. All participants of the groups were evaluated with the Zung Self-Rating Anxiety Scale (SAS) [22] and the Zung Self-Rating Depression Scale (SDS) [23]. Forty healthy age- and gender-matched controls were enrolled from the local area by poster advertisement. The controls were free of a history of any form of headache in addition to referring to the exclusion criteria of ETTH. The study protocol was approved by the local ethics committee. Prior to study inclusion, all participants received a complete description of the study and granted written informed consent according to the Declaration of Helsinki.

### Brain MRI acquisition and analysis

#### Image acquisition

High-resolution T1-weighted images, using a 3D-spoiled gradient echo sequence were obtained on a 3.0-Tesla MRI scanner (Siemens Verio, Erlangen, Germany) with a standard head coil for all participants. The scanning sequences were as follows: repetition time (TR) = 8.5 ms; echo time (TE) = 3.93 ms; flip angle = 12°; slice thickness = 1 mm; field of view (FOV) = 240 × 240 mm^2^; matrix size = 256 × 256; in-plane resolution = 0.47 × 0.47 mm^2^; and number of slices = 156. A T2-weighted axial scan and a coronal fluid-attenuated inversion recovery (FLAIR) scan were also acquired to exclude brain lesions in patients and controls.

#### Preprocessing

For cross-sectional data, image pre-processing was performed with Statistical Parametric Mapping version 8 (SPM8) (www.fil.ion.ucl.ac.uk/spm) using the VBM toolbox with Diffeomorphic Anatomic Registration Through Exponentiated Lie Algebra (DARTEL) on the Matlab 10.0 platform (The Mathworks, Natick, MA, USA). Images were initially assessed for scanner artifacts and gross anatomical abnormalities for each subject. Then, the anterior commissure was set as the origin of spatial coordinates along the reoriented anterior–posterior commissure line. All imaging analyses were conducted as suggested by the VBM Tutorial (http://www.fil.ion.ucl.ac.uk/~john/misc/VBMclass10.pdf). The process is briefly summarized as follows: (1) The T1-weighted images were segmented into GM, white matter (WM) and non-brain voxels (cerebrospinal fluid, skull) using the ‘new-segment’ routine implemented in SPM8 [24]. (2) Population templates (GM, WM) were generated from the entire image dataset using the DARTEL algorithm [25, 26]. (3) All images were normalized into Montreal Neurological Institute (MNI) stereotactic space with the normalized images modulated to correct volume changes by the Jacobian determinants. (4) Images were smoothed by convolution with an isotropic Gaussian kernel of 8-mm full-width at half maximum before statistical analyses.

For longitudinal imaging data (pain ETTH vs. pain-free ETTH), we conducted the VBM analysis using the Computational Anatomy Toolbox (CAT12) (http://www.neuro.uni-jena.de/cat12/CAT12-Manual.pdf). Image pre-processing used the default settings involving intra-subject realignment, bias correction, segmentation, and normalization. A flexible factorial model was applied for the statistical analysis in one group with 2 time points.

### Statistical analyses

For demographic and clinical data, the Statistical Package for the Social Sciences software version 19.0 (SPSS Inc., Chicago, IL) was used for statistical evaluation. Continuous variables were examined using 2-tailed paired or 2-sample *t* tests. For categorical data, χ^2^ tests were applied.

For cross-sectional GM density (GMD) analysis, a general linear model in SPM8 was applied within and between groups (pain-free ETTH vs. healthy controls; pain ETTH vs. healthy controls) to assess the possible morphological changes with covariation for the age, the interval time between scans, total intracranial volume (TIV), and SAS and SDS scores. For longitudinal GMD analysis (pain ETTH vs. pain-free ETTH), a flexible factorial model was used. The statistical significance level was set at *p* < 0.05, corrected by AlphaSim (per-voxel *p* < 0.001 with cluster size greater than 33 contiguous voxels). We performed further analyses to explore the correlation between regional GMD over the entire brain and clinical features (disease duration, headache days per month, VAS score, SAS score, and SDS score) in ETTH patients with age and TIV as covariates. A *p* value less than 0.05 after correction for multiple comparisons was considered significant.

## Results

### Clinical data

All participants completed the study. Table 1 summarizes the demographic and clinical data of study participants. There were no significant differences in sex, age, education level, or handedness between ETTH patients and healthy controls (all *p* > 0.05). The interval time between scans in ETTH patients was 6.56 (2.72) days. Compared with healthy controls, ETTH patients had significantly higher scores on the SAS and SDS for both in pain and out of pain phases (all *p* < 0.05). The SAS and SDS scores in ETTH patients were significantly increased during the pain phase compared with the pain-free phase (both *p* < 0.05).

**Table 1:** Demographic variables and clinical characteristics of study participants

Demographic variables	ETTH patients (during attacks)	ETTH patients (interictal period)	Healthy controls	*p* Value
Age (years)	35.00 (9.27)	35.00 (9.27)	34.48 (6.94)	0.78
Sex (male/female)	19/21	19/21	20/20	0.82
Handedness (left/right)	2/38	2/38	2/38	1.00
Education (years)	11.23 (3.05)	11.23 (3.05)	11.45 (3.13)	0.54
Disease duration (years)	5.50 (3.15)	5.50 (3.15)	–	–
Headache days per month	5.30 (3.00)	5.30 (3.00)	–	–
VAS score (0–100)	48 (9.53)	–	–	–
SAS score	54.13 (5.42)	43.65 (4.77)	27.50 (6.73)	<0.001*
				<0.001**
				<0.001***
SDS score	40.25 (6.06)	34.83 (5.62)	28.7 (6.42)	<0.001*
				<0.001**
				<0.001***

Values are expressed as mean (SD). The *p* values were calculated using appropriate statistical tests (2-tailed paired *t* test or 2-sample *t* test for continuous data and χ^2^ tests for categorical data)

*ETTH* episodic tension-type headache, *SAS* self-rating anxiety scale, *SDS* self-rating depression scale, *VAS* visual analog scale

*2-tailed paired t test for SAS or SDS scores between pain phase and out of phase in ETTH patients

**2-sample t test for SAS or SDS scores between ETTH patients in pain phase and healthy controls

***2-sample t test for SAS or SDS scores between ETTH patients out of pain phase and healthy controls

### Whole-brain VBM data

**Fig. 1:**
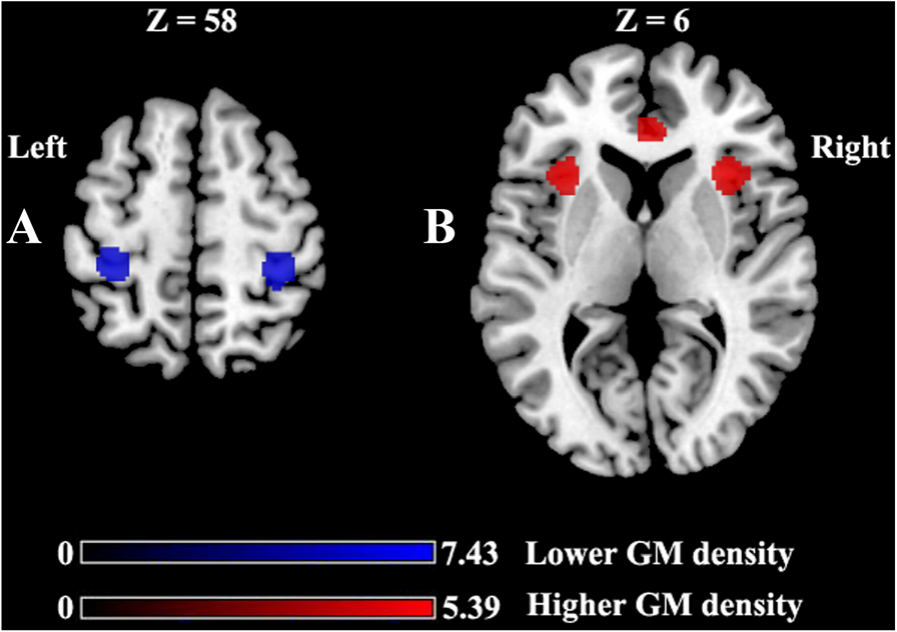
**a**: lower GM density in the bilateral primary somatosensory cortex, **b**: higher GM density in the bilateral anterior cingulate cortex and anterior insula

No significant differences were identified between patients and controls for the total volume of GM, WM, or TIV. As demonstrated in Table 2 and Fig. 1, significant GMD reductions in the bilateral primary somatosensory cortex (S1) (A) and significant GMD increases in the bilateral anterior cingulate cortex (ACC) and the bilateral anterior insula (B) were observed between the pain phase and pain-free phase in patients with ETTH. Compared to healthy controls, patients with ETTH in the out of pain phase exhibited similar GMD changes. In contrast, the ETTH patients out of pain phase showed no region with higher or lower GMD compared with healthy controls. Furthermore, the whole brain correlation analyses revealed that GMD in the left ACC and left anterior insula was negatively correlated with headache days per month (*r* = −0.782, *p* = 0.002 and *r* = −0.646, *p* = 0.007, respectively). In addition, GMD in the left ACC was negatively correlated with the SAS score (*r* = −0.841, *p* = 0.001) and the SDS score (*r* = −0.579, *p* = 0.021) in ETTH patients during the pain phase. No correlation was identified between regional GMD and disease duration or regional GMD and the VAS score in ETTH patients.

**Table 2:** Summary of gray matter density differences in ETTH patients between the pain and pain-free phases

Brain regions	Brodmann areas	Maximum MNI coordinates (x, y, z)	Voxels	T value
Pain phase < pain-free phase				
Right primary somatosensory cortex	3/4	32,–36, 62	300	7.43
Left primary somatosensory cortex	3/4	−34, −34, 58	155	6.61
Pain phase > pain-free phase				
Bilateral anterior cingulate cortex	32/24	10, 38, 16	493	5.35
Left anterior insula	13	34, 20, 6	255	5.39
Right anterior insula	13	−34, 21, 7	218	5.35

*ETTH* episodic tension-type headache, *MNI* Montreal Neurological Institute

## Discussion

To the best of our knowledge, this is the first longitudinal study that primarily investigated whether treatment-naïve ETTH patients have dynamic changes of brain GMD in different pain states. Our results demonstrated a lower GMD in the bilateral S1 and a higher GMD in the bilateral ACC and anterior insula in ETTH patients during the pain phase compared with the pain-free phase. In contrast, no GMD changes were observed in ETTH patients during the pain-free period. Our study exhibited a dynamic cortical plasticity in patients with ETTH. Furthermore, the correlation analyses indicated that these GMD changes could be affected by the headache days per month and anxiety and depressive symptoms.

Convergent evidence from anatomical, imaging, and lesion data reveals that the S1, the ACC, and the anterior insula are key regions implicated in complex nociceptive processing [27, 28]. The S1 is responsible for detecting the presence and magnitude of a pain stimulus and is involved in pain perception [27]. The ACC participates in the emotional-motivational aspect of pain [29]. Often working together with the ACC, the anterior insula is proposed to involve in the integration of polymodal sensory information as well as the integration of emotional and cognitive processes [29, 30]. A recent neuroimaging meta-analysis revealed common activations during pain for healthy subjects and patients with chronic pain in the ACC and the anterior insula regardless of modality, body part, or clinical experience [29]. This finding further supported the central role of the ACC and the anterior insula in human pain processing [29]. Painful stimulation during the pain phase in ETTH patients contribute to these brain functional changes, which could further lead to the structural reorganization observed in our study.

Of note, the structural abnormalities were not observed in the pain-free period in ETTH patients. This structural reorganization and dynamic change may be ascribed to the transmission of sensory input and pain perception [31]. Although little is known regarding the neurobiological basis of this dynamic pattern in ETTH, fast adjusting reversible neuronal processes, such as dendrite spine and synapse turnover, are more likely responsible for these rapid morphometric changes [9]. This feature may reflect a defensive adaptation designed to orient cortical attention towards stimuli that threaten the body’s integrity [31, 32] or reflect a balance of descending pain modulatory circuits [31, 33, 34].

However, this adaptation or balance might be disrupted as the headache days increased and anxiety and depressive symptoms progressed. Our correlation analyses demonstrated that ETTH patients with longer headache days per month had lower GMD in the ACC and anterior insula in our study. Increased headache days may contribute to the progression from episodic to chronic TTH, which leads to the central sensitization with GM reductions in multiple cerebral regions, such as the ACC and the anterior insula [17]. This correlation also contributes to explaining why we observed increased GMD in the ACC and anterior insula in ETTH not like that most studies reported decreases here in chronic pain diseases, including chronic TTH [16, 35]. Increased GMD in ETTH may reflect a defensive adaptation, whereas decreased GMD may indicate decompensation as disease develops to the chronic form. This dynamic change in these areas calls on us to pay more attention to TTH in the episodic form. In addition, anxiety and depressive symptoms are common in ETTH patients [36]. The comorbidity may confer a worse prognosis in TTH patients [36, 37]. Although their pathophysiology remains unknown, their relationship may be bidirectional [36]. ACC is the common neuroanatomical site implicated in mental illness, including depression, anxiety and other psychiatric disorders [38]. Our data demonstrated negative correlations between GMD in the ACC and the SAS and SDS scores. This information calls attention to a timely recognition of these symptoms and the need to offer proper treatment in ETTH patients.

Some limitations should be mentioned when interpreting the findings of our study. First, VBM has inherent limitations. For example, VBM detects only linear, spatially limited differences [39]. Second, our study did not investigate a control group longitudinally in the same time intervals using the same preprocessing and statistics, although the interval time was short, which might bias our results. Third, this study is the first to evaluate the brain structural changes in ETTH; further studies would benefit from integrating both structural and functional networks associated with the pathophysiological underpinnings of ETTH.

## Conclusions

This is the first study to demonstrate dynamic and reversible GMD changes in the S1, ACC, and anterior insula between pain and pain-free phases in ETTH patients, which suggests cerebral adaptation to pain stimuli with a balance of pain modulatory circuits. However, this balance might be disrupted by increased headache days and progressive anxiety and depressive symptoms. Future studies are warranted to determine whether this structural plasticity is a characteristic of ETTH.

## Abbreviations

ACC: Anterior cingulate cortex
DARTEL: Diffeomorphic Anatomic Registration Through Exponentiated Lie Algebra
ETTH: Episodic tension-type headache
FOV: Field of view
GM: Gray matter
GMD: Gray matter density
ICHD: International Classification of Headache Disorders
MNI: Montreal Neurological Institute
S1: Primary somatosensory cortex
SAS: The Zung Self-Rating Anxiety Scale
SDS: The Zung Self-Rating Depression Scale
SPM8: Statistical Parametric Mapping version 8
SPSS: Statistical Package for the Social Sciences
TE: Echo time
TIV: Total intracranial volume
TR: Repetition time
TTH: Tension-type headache
VAS: Visual analog scale
VBM: Voxel-based morphometry
WM: White matter
